# EXPERIENCES WITH APATHY AND MHEALTH PREFERENCES AMONG ADULTS WITH MILD COGNITIVE IMPAIRMENT

**DOI:** 10.1093/geroni/igac059.1480

**Published:** 2022-12-20

**Authors:** Pilar Ingle, Evan Plys, Danielle Kline, Emma Stark, Jennifer Dickman Portz

**Affiliations:** University of Denver, Lakewood, Colorado, United States; University of Colorado School of Medicine, Aurora, Colorado, United States; University of Colorado School of Medicine, Aurora, Colorado, United States; University of Colorado School of Medicine, Aurora, Colorado, United States; University of Colorado Anschutz, Aurora, Colorado, United States

## Abstract

Apathy is common early in dementia, often increases in severity as illness progresses, and is one of the most pervasive neuropsychiatric symptoms associated with Alzheimer’s Disease and related dementias (ADRDs). This study explored experiences of apathy and preferences for dyadic-communication via mobile health (mHealth) among adults with mild cognitive impairment (MCI) and their care-partners. Semi-structured interviews were conducted with 10 ADRD-related health providers (5 behavioral neurologists, 2 nurse practitioners, 2 geriatricians, 1 nurse, 1 social worker), 9 patients with MCI (5 male, mean age = 73), and 6 of their care-partners. Participants expressed interest in the development of mHealth dyadic communication tools targeting apathy symptom monitoring, dyadic understanding of apathy, spiritual and religious coping, sharing ADRD health information, and resources for addressing burdensome behavioral symptoms of MCI. This research provides preliminary insight to the development of apathy specific mHealth communication strategies that may improve patient and care-partner quality of life.

